# Associations between apparent temperatures and emergency ambulance calls in Wuxi, China: a time series analysis

**DOI:** 10.3389/fpubh.2025.1652961

**Published:** 2025-09-09

**Authors:** Chao Yang, Xiuzhu Li, Wanjun Zhang, Yiru Tao, Pengfei Zhu, Chuncheng Lu, Weijie Zhou, Xinliang Ding

**Affiliations:** ^1^The Affiliated Wuxi Center for Disease Control and Prevention of Nanjing Medical University, Wuxi Center for Disease Control and Prevention, Wuxi Medical Center, Nanjing Medical University, Wuxi, China; ^2^Statistics and Information Center of Wuxi Health Commission, Wuxi, China; ^3^Key Laboratory of Modern Toxicology of Ministry of Education, School of Public Health, Nanjing Medical University, Nanjing, China

**Keywords:** apparent temperature, emergency ambulance calls, extreme temperatures, attributable risk, time series analysis

## Abstract

Inappropriate apparent temperature (AT) is a major global threat to human health. Although the association between temperature and health has been studied extensively, limited evidence regarding emergency ambulance calls (EACs) is available. Daily emergency department visit records, meteorological data, and air pollutant data were obtained from 1 January 2014 to 31 December 2019. A distributed lag non-linear model was applied to examine the interaction between AT and lagged effects. The analysis was stratified according to disease aetiology, sex, and age. The proportion of EACs attributable to AT was calculated as an indicator of associated burden. A non-linear U-shaped relationship was observed between AT and non-accidental, cardiovascular, and circulatory EACs, with the lowest risk AT occurring at 22.54 °C, 17.12 °C, and 12.74 °C, respectively. High AT effects manifested immediately, whereas low AT effects were delayed. Stratified analysis indicated that males and individuals aged ≥65 years demonstrated heightened sensitivity to both extreme heat (97.5th percentile, 35.98 °C) and cold (2.5th percentile, −1.27 °C), whereas females displayed greater vulnerability to extreme heat [relative risks = 1.16, 95% confidence intervals (CI): 1.04–1.29]. The attributable fraction (AF) for non-accidental EACs in Wuxi was 6.88% (95% CI: 4.09–9.26%), with higher AFs observed for cardiovascular (10.37%) and respiratory (4.94%) emergencies. Moderate thermal variations substantially affected the EACs more than extreme AT conditions. These findings underscore the necessity of implementing early warning mechanisms targeting thermal extremes and developing temperature-regulated public facilities to safeguard vulnerable groups, particularly older citizens, during extreme temperature events.

## 1 Introduction

Global climate change has increased the number of extreme weather events and significantly affected public health. Heatwaves, intensified by human activity, cause over one-third of global heat-related deaths, primarily from cardiovascular and respiratory diseases (1–3). Annually, 1.8 million cardiovascular deaths (CVDs) constitute 8.86% of total CVDs and are temperature-related (4).

Temperature-cardiorespiratory relationships typically follow U-, V-, or J-shaped curves, indicating increased cardiopulmonary risk at temperature extremes (5–7). These non-linear relationships manifested differently across climatic regions. For example, in Mediterranean climates, such as Spain, extreme heat elevates mortality risks from hypertension, heart failure, and pneumonia (8). Summer heat accounts for 16.2 and 22.3% of respiratory deaths in Madrid and Barcelona, respectively (9). Globally, non-optimal temperatures account for 8.86% of CVDs, with increased heat-related mortality (4). In Beijing, extreme cold showed prolonged effects (21 days) on respiratory-related emergency visits, whereas extreme heat had immediate effect (7 days), particularly affecting older males with respiratory conditions (10).

Existing research predominantly relies on mortality and hospitalisation data (11, 12). These outcomes fail to detect early-stage temperature-related symptoms that precede fatal events. Mortality-based assessments suffer from delayed reporting and capture only a subset of temperature-health impacts. Hospitalisation records face challenges in timely access and precise symptom onset determination. In contrast, data from emergency ambulance calls (EACs) integrate regional health indicators with ambulance dispatch records, demonstrating superior temporal sensitivity in identifying acute disease onset (13).

Several studies have assessed the association between extreme temperatures and EACs; however, the results of these studies are conflicting. One study demonstrated that CVD-related EACs did not significantly increase at high temperatures (14). In contrast, another study revealed that a 5 °C increase in average temperature was associated with a 2% increase in all-cause CVD EACs (15). In addition, the association between extreme temperatures and EACs varies according to sex (16) and age (49). The lag effects of the EACs caused by extreme temperatures are also inconsistent. The results of a meta-analysis showed that the combined risk of all ambulance calls peaked at a lag of 16–18 h when hourly temperatures were in the 99th percentile (50). A single temperature indicator may not be sufficient to describe the true relationship because human comfort depends on temperature and other meteorological factors, such as humidity and wind speed. Therefore, it is necessary to use composite indicators of temperature along with other meteorological factors when studying the effects of temperature on health. Apparent temperature (AT), as a composite index of ambient temperature, wind speed, and humidity, reflects the thermal sensation of the human body more objectively than the temperature itself (17). Previous studies on the relationship between AT and EACs have focused on Europe (18, 19), whereas few have focused on Asia. This integrated approach addresses a key limitation in prior studies using singular meteorological parameters, particularly relevant in humid subtropical climates such as that in Wuxi, where summer relative humidity routinely exceeds 80% (20).

In this study, we evaluated the association between non-optimal AT and non-accidental EACs, including cardiovascular and respiratory EACs, using records from Wuxi City between 2014 and 2019. Furthermore, we examined the potential effect of modifications by sex and age on the AT-EACs relationship. The analytical pipeline integrates distributed lag non-linear modelling (DLNM) with attributable fraction (AF) calculations, using a three-stage approach: (1) Exposure-response curve estimation; (2) Lag-effect decomposition; (3) Population-attributable risk stratification. These findings will directly inform the development of tiered early warning systems in the Yangtze Delta megalopolis.

## 2 Materials and methods

### 2.1 Study area

Wuxi is geographically located in the southern region of Jiangsu Province, China, within the core of the Yangtze River Delta. The city spans from approximately 31°33’N to 32°08’N latitudes and 119°48′E to 120°23′E longitudes. Situated between the Yangtze river and Taihu lake, Wuxi is characterised by predominantly flat terrain with relatively low elevation. The region exhibits a typical subtropical humid monsoon climate characterised by four distinct seasons, including hot humid summers and cold damp winters.

### 2.2 EAC data

Hourly EAC data for Wuxi, from 1 January 2014 to 31 December 2019, were obtained from the Wuxi Emergency Center. The dataset encompasses comprehensive patient-related information, including the timestamp of the call, geographic location, demographic characteristics (age and sex), provisional diagnosis, and associated clinical symptoms. All emergency calls initiated during the study period were documented systematically. To ensure data confidentiality, sensitive personal identifiers, such as patient names, residential addresses, and contact numbers, were encrypted before data extraction. Subsequently, EACs attributable to natural causes were extracted, whereas those associated with external events, including accidents, poisoning, and obstetric emergencies, were excluded from the final analysis. Additionally, records lacking date information or originating outside the jurisdictional boundaries of Wuxi City were omitted to maintain data integrity. Furthermore, primary diagnoses were categorised based on the International Classification of Diseases, Tenth Revision (ICD-10), with particular emphasis on conditions related to the cardiovascular (I00–I99) and respiratory (J00–J99) systems to enable targeted analysis.

### 2.3 Meteorological and air pollutants data

Air pollutant data were collected from a network of nine monitoring stations operated by the Wuxi Municipal Ecological Environment Bureau. The dataset included the daily average concentrations of six major air pollutants: fine particulate matter (PM_2.5_), respirable particulate matter, sulfur dioxide, nitrogen dioxide, carbon monoxide, and ozone (O_3_), from January 2014 to December 2019.

Meteorological data, including average temperature, relative humidity, and wind speed, were obtained from daily monitoring records provided by the Wuxi Meteorological Bureau from January 2014 to December 2019. AT was calculated as an exposure variable based on these three meteorological parameters following established methodologies (18, 21, 22) (Equations 1 and 2).


(1) AT=−2.7+1.047T+0.2Pv−0.65w

(2) Pv=(rh/100)6.1094e^((17.625T/(243.04+T)))

where *T* is the average temperature (°C), *Pv* is the vapour pressure (hPa) estimated using formula (2), *w* is the wind speed (m/s) 10 m above the ground, and *rh* is the relative humidity (%).

### 2.4 Statistical analysis

To evaluate the distributed lag effect of AT on EACs, a quasi-Poisson generalised additive model (GAM) integrated with a DLNM was used. Through the application of “cross-basis” functions, DLNM can effectively capture both the non-linear exposure-response relationship and lagged effects. The structure of the constructed model was as follows (Equation 3) (23):


(3) $$\begin{array}{l} \log[E(\mathrm{Yt})]=\alpha +\mathrm{cb}(\text{Tempt},l,\mathrm{lag}=14)+\mathrm{ns}(\text{time},\frac{7}{\mathrm{per}}\text{year})\\ +\mathrm{ns}(\text{humidity},3)+\mathrm{ns}(\text{pollutants},3)+\beta \;\mathrm{DOW}+\gamma \;\text{Holiday} \end{array}$$

where Yt is the EACs; *α* is the intercept; β and γ are regression model coefficients of week and holiday variables, respectively; *cb*(*Tempt,l*) refers to the cross-basis matrix with AT and lag days; *ns* is the natural cubic spline function; *humidity* and *pollutants* represent relative humidity and air pollutants, both included in the natural cubic spline function with degrees of freedom (*df*) equal to 3; *time* is the time variable to control for long-term trend; *Holiday* is the Chinese public holidays; *DOW* denotes the day of week.

This study determined the maximum number of lag days to be 14 days, with *df* for relative humidity and time being 3 and 7, respectively. The optimal model based on Akaike Information Criterion (AIC) index selected O_3_ and PM_2.5_ to be included in the model.

Consistent with the established concept of minimum mortality temperature in prior research (24), the AT associated with the lowest risk of EACs was identified as the optimal AT in this study. Extremely high and low ATs were operationally defined as ATs corresponding to the 2.5th and 97.5th percentiles, respectively (25). The optimal lag period for AT was determined to be 14 days based on the AIC values derived from the lag days model. To elucidate the exposure-response relationship, we quantified the associations between specific AT thresholds (2.5th, 5th, 7.5th, 10th, 90th, 92.5th, 95th, and 97.5th percentiles) and EACs. AT-related effects were expressed as relative risks (RRs) with corresponding 95% confidence intervals (CIs). In addition, further exploration of potential variations in risk was conducted using a subgroup analysis stratified by sex and age.

The total AF was calculated to estimate the cumulative effects of non-optimal AT on the EACs, using the optimum AT as the reference point. The formula is expressed as follows (Equation 4):


(4) $$\mathrm{AFs}=\sum_{i=l0}^{L}\frac{\left(\mathrm{RRi}-1\right)}{(\mathrm{RRi}-1)+1}=1-\exp(-\sum_{i=l0}^{L}\mathrm{\beta xi})$$

where *RRi* is the relative risk at each exposure level compared to the baseline level, *l_0_* and *L* represent the minimum and maximum lag days, respectively, βxi is the effect parameter at exposure level *i*.

Using optimum AT as the central reference point, four distinct thermal exposure categories were established (26): extreme cold (AT ≤ P_2.5_), moderate cold (P_2.5_ < AT < minimum AT), moderate heat (minimum AT < AT < P_97.5_) and extreme heat (AT ≥ P_97.5_). To quantify the uncertainty associated with A*F* estimates, Monte Carlo simulation methods were used to derive 95% empirical confidence intervals. Demographic stratification analyses were subsequently performed across sex and age groups (0–65 vs. 66 years) to assess the differential vulnerability patterns in temperature-associated morbidity risks (27).

All statistical analyses were conducted using the “mgcv” and “dlnm” software packages in R version 4.3.2.

### 2.5 Sensitivity analysis

To evaluate the robustness of the model, a sensitivity analysis was performed by assessing alternative lag periods (7 and 21 days), adjusting the degrees of freedom for time trends (range, 6–9) and relative humidity (range, 4–6). Additionally, potential confounding by NO_2_ and SO_2_ was evaluated through separate pollutant-adjusted models.

## 3 Results

### 3.1 Descriptive analysis

Table 1 presents the baseline information on daily EACs, meteorological factors, and air pollutants in Wuxi from 2014 to 2019, covering 2,191 days. During the study period, 141,031 EAC records were collected, averaging 55.15 ± 14.1 EACs daily. Among these records, approximately 54.6% were males, and 45.4% were females. The proportion of individuals aged 65 and above was higher than those aged 0–65 years (58.62% vs. 41.38%). The average daily AT was 16.53 ± 8.93 °C, slightly lower than the average ambient temperature (17.35 ± 8.9 °C). During the study period, the meteorological conditions were characterised by the following daily averages: relative humidity was 73.28% (±13.32%), wind speed averaged 2.16 m/s (±0.83 m/s), O_3_ concentration was 100.19 μg/m^3^ (±49.98 μg/m^3^), and PM_2.5_ concentration averaged 49.11 μg/m^3^ (±30.2 μg/m^3^).

**Table 1:** Descriptive statistics for daily EACs, meteorological variables and air pollutants from 2014 to 2019 in Wuxi, China.

Variable	Mean ± SD	Min	P_25_	Median	P_75_	Max
Non-accidental EACs	55.15 ± 14.1	6	49	56	64	112
Male	30.86 ± 8.82	2	26	31	36	61
Female	24.3 ± 7.23	1	20	25	29	55
0 ~ 65 years	21.68 ± 6.62	1	18	22	26	53
65 ~ years	33.48 ± 9.88	3	28	34	39	69
Cardiovascular EACs	10.32 ± 4.37	2	7	10	14	19
Male	5.41 ± 2.42	0	4	5	7	14
Female	4.92 ± 2.4	0	3	5	7	11
0 ~ 65 years	3.45 ± 2.24	0	2	3	5	9
66 ~ years	6.92 ± 2.3	2	5	7	9	15
Respiratory EACs	8.55 ± 2.36	3	7	9	10	18
Male	4.51 ± 1.61	0	3	5	6	12
Female	4.03 ± 1.56	0	3	4	5	8
0 ~ 65 years	2.53 ± 1.34	0	2	3	4	6
66 ~ years	6.03 ± 1.42	2	5	6	7	14
PM_2.5_ (μg/m^3^)	49.11 ± 30.2	6.8	28.1	41.6	61.9	238.4
O_3_ (μg/m^3^)	100.19 ± 49.98	0	61.4	90	132.8	275.9
Relative humidity (%)	73.28 ± 13.32	27	64	74	83	100
Ambient temperature (°C)	17.35 ± 8.9	−6.1	9.5	18	24.6	36
Apparent temperature (°C)	16.53 ± 8.93	1.51	8.63	14.8	24.11	37.41
Wind speed (m/s)	2.16 ± 0.83	0.3	1.6	2.1	2.6	6.7

EACs, emergency ambulance calls; SD, standard deviation; P_25_ = the 25th percentile; P_75_ = the 75th percentile; PM_2.5_, particulate matter (with aerodynamic diameter) less than 2.5 micrometers; O_3_, Ozone.

Average ambient temperature, relative humidity, wind speed, AT, PM_2.5_ and O_3_ exhibited seasonal variations. The average ambient temperature, AT, and O_3_ trends were similar, maximizing maximum in summer and minimizing in winter. In contrast, PM_2.5_ peaked in winter and showed the opposite trend (Supplementary Figure S1).

### 3.2 Overall effects of AT on EACs

**Figure 1:**
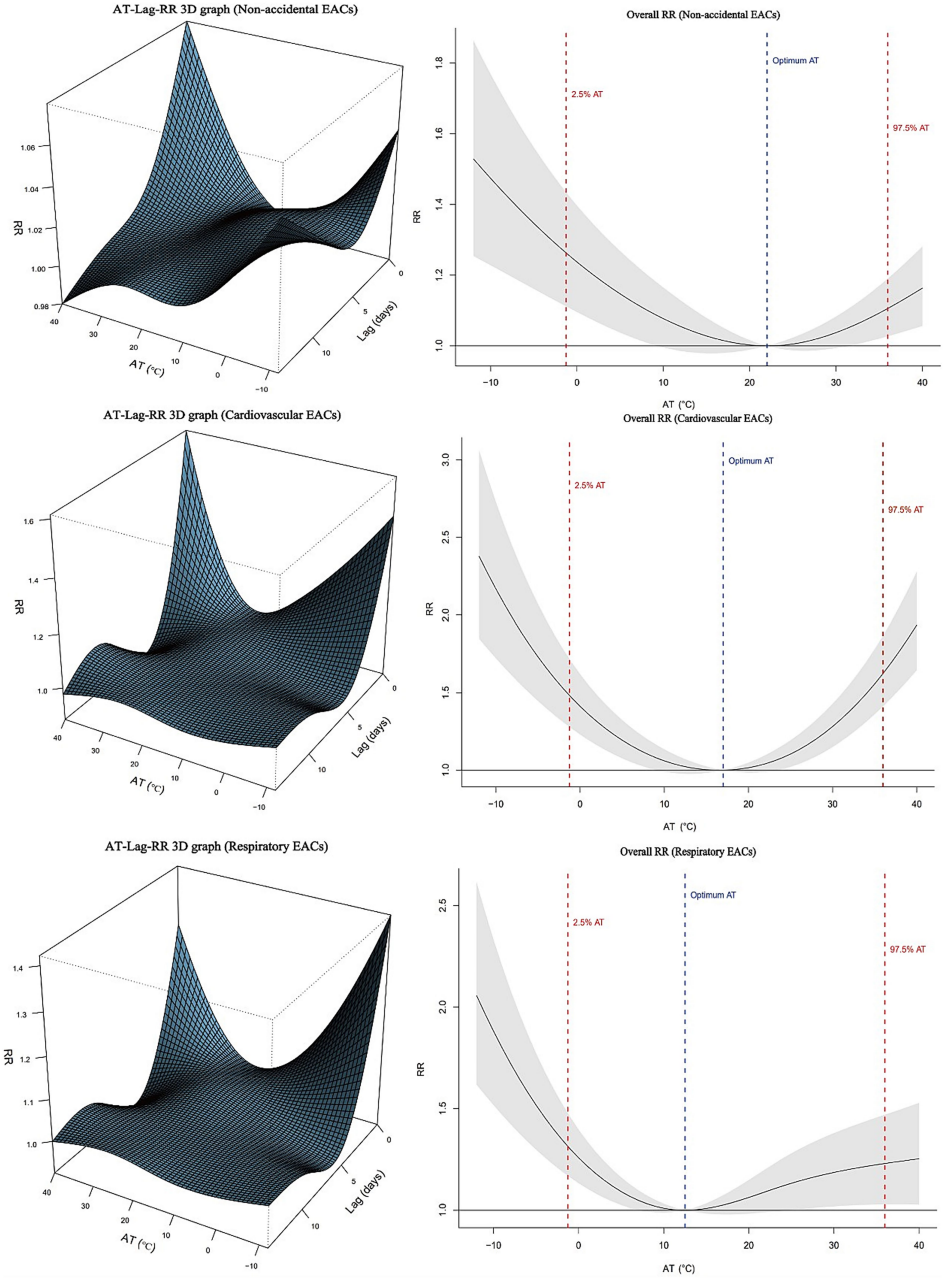
Association between apparent temperature (AT) and emergency ambulance calls (EACs) in Wuxi, 2014–2019. Left panels: Three-dimensional exposure-lag-response risk surfaces. Right panels: Overall relative risks with 95% confidence intervals.

Figure 1 presents the overall exposure-response relationships between AT and EACs, along with the lag effects in the population. The calculated optimal ATs were 22.54 °C, 17.12 °C and 12.74 °C for non-accidental, cardiovascular and respiratory EAC, respectively. Both extreme cold (2.5th percentile) and extreme heat (97.5th percentile) demonstrated significant risk effects on EACs (Supplementary Tables S1–S3).

**Figure 2:**
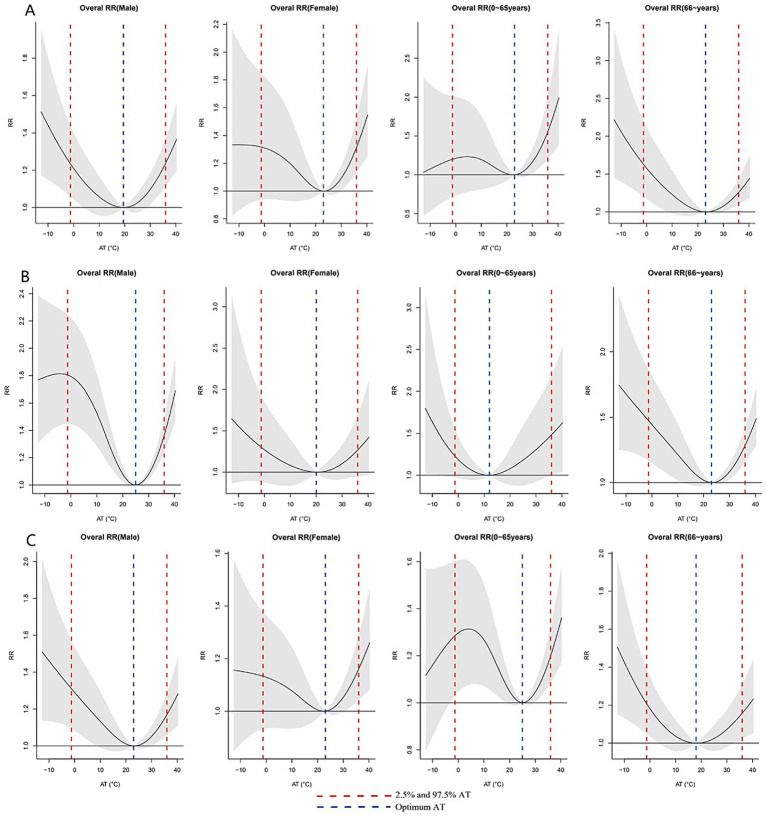
Exposure–response relationships between AT and EACs across subgroups, referenced to group-specific optimum AT values. **(A)** Non-accidental EACs; **(B)** Cardiovascular EACs; **(C)** Respiratory EACs. Wuxi, China 2014–2019.

Figure 2 illustrates the exposure-response relationship between AT and EACs across different subgroups with reference to the optimal AT. With an increase in AT degree polarisation (cold: 10th, 7.5th, 5th, and 2.5th percentiles; heat: 90th, 92.5th, 95th, and 97.5th percentiles), the risks of non-accidental, cardiovascular, and respiratory EACs showed an upward trend. For males and individuals aged >65 years, both extreme heat and cold exhibited significant associations with non-accidental, cardiovascular, and respiratory EACs. In females, no statistically significant associations were observed between extreme cold and any EAC category (*p* > 0.05), whereas extreme heat significantly increased risks of non-accidental (RR = 1.16, 95% CI: 1.04–1.29) and respiratory EACs (RR = 1.31, 95% CI: 1.13–1.52). Among the 0–65 year age group, extreme cold showed significant respiratory EAC risks (RR = 1.80, 95% CI: 1.45–2.25), whereas extreme heat was associated with elevated risks of non-accidental (RR = 1.36, 95% CI: 1.24–1.49) and respiratory EACs (RR = 1.47, 95% CI: 1.17–1.86). Notably, cardiovascular EACs demonstrated no significant temperature-associated risks in the 0–65 year age group (Supplementary Tables S1–S3).

### 3.3 Delayed effects of AT on EACs

**Figure 3:**
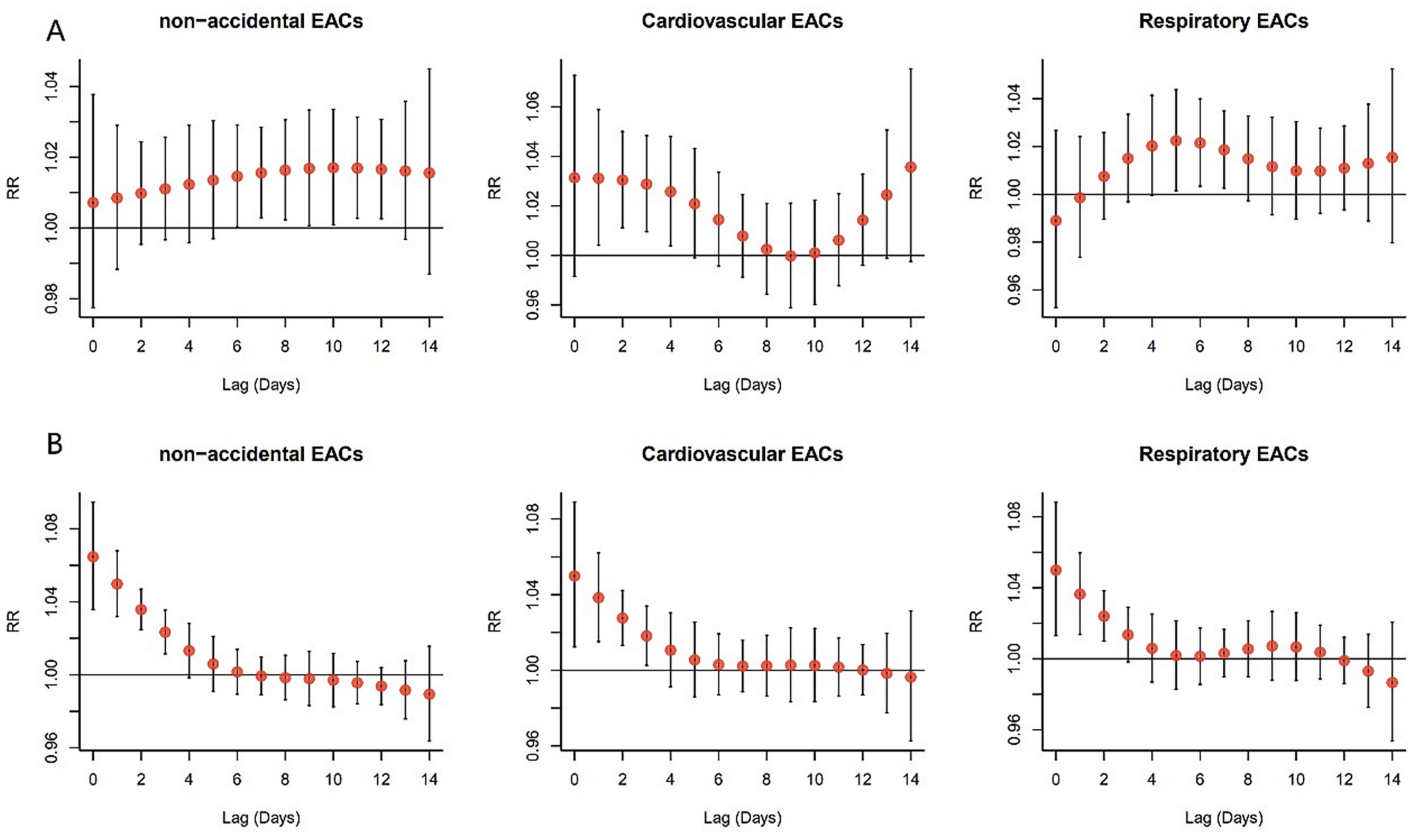
Lagged effects of extreme AT percentiles on EACs, referenced to group-specific optimum AT. **(A)** Extreme cold (2.5th percentile); **(B)** Extreme heat (97.5th percentile). Wuxi, China 2014–2019.

Figure 3 shows the individual effects of AT on non-accidental, cardiovascular, and respiratory EACs over different lag days. The effect of extreme cold (2.5th percentile) varied across different types of EACs. For non-accidental EACs, significant effects were observed from lag 7 to lag 12, with the maximum effect occurring at lag 12 (RR = 1.017; 95% CI: 1.003–1.031). For cardiovascular EACs, significant effects were noted from lag 1 to lag 4, with the maximum effect observed at lag 1 (RR = 1.031, 95% CI: 1.004–1.059). Similarly, for respiratory EAC, significant effects were observed from lag 5 to lag 7, with the largest effect occurring at lag 5 (RR = 1.022, 95% CI: 1.003–1.048). The impact of extreme heat followed a similar pattern, with the maximum effect occurring on the first day, gradually diminishing and becoming negligible after 4 or 5 days (Supplementary Table S4).

**Figure 4:**
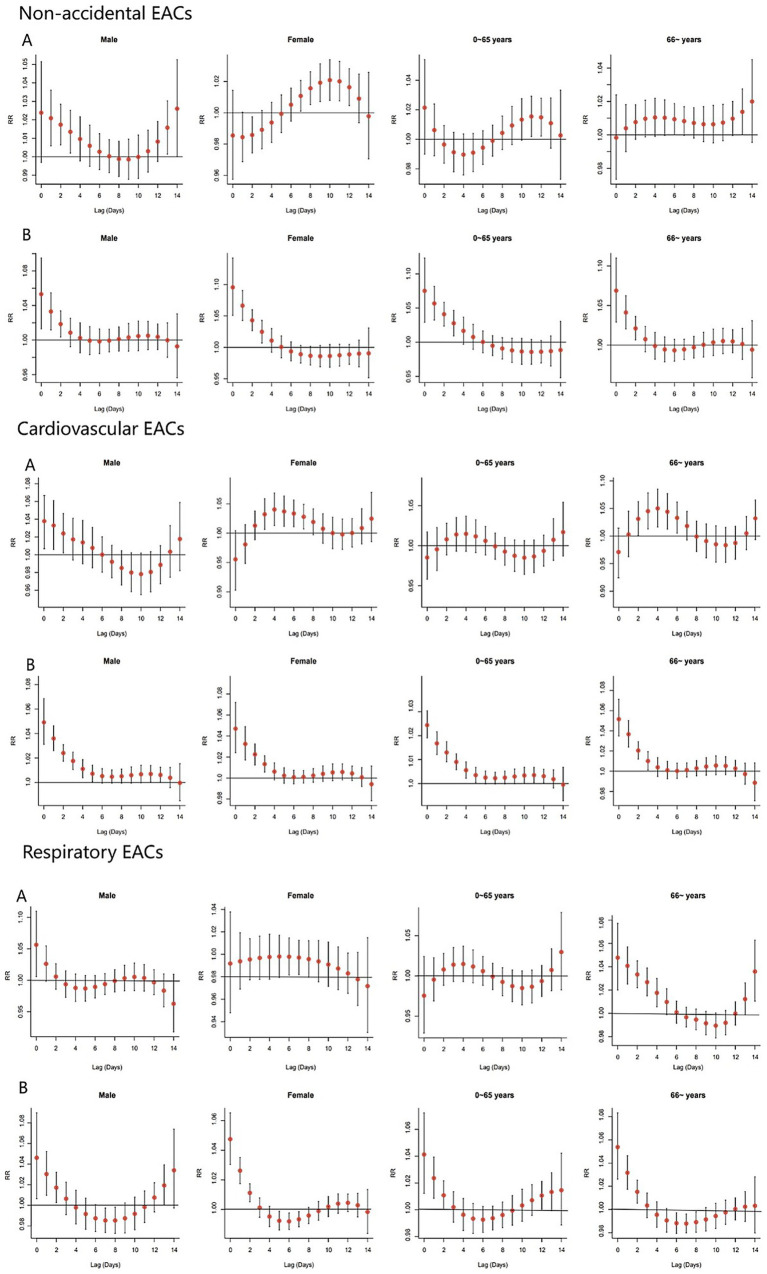
Single-day lag effects of AT on EACs by cause type. Reference temperatures: **(A)** −1.27 °C (cold effect); **(B)** 35.98 °C (heat effect). Wuxi, China 2014–2019.

Figure 4 presents the single-lag effects for extreme cold (2.5th percentile) and heat (97.5th percentile) in the non-accidental, cardiovascular, and respiratory EACs subgroups. For extreme cold, the lag effects of non-accidental, cardiovascular, and respiratory EACs were similar within the same sex; the effect on males was predominantly acute, peaking on the first day, and subsequently attenuating. In contrast, females exhibited varying lag patterns, with significant effects observed at lag 7–12, 3–7, and 6–7. In the 0–65 year age group, non-accidental EACs showed an effect at lag 11–12, whereas no significant differences were observed for cardiovascular and respiratory EACs. Among those aged ≥65 years, cardiovascular EACs were influenced at lag 3–6, whereas respiratory EACs exhibited effects at lag 0–4, with the maximum impact occurring on the first day. The effects of extreme heat exposure on sex and age were similar. The largest risk effect occurred at lag 0 days and gradually diminished to no significance with an increase in lag days.

### 3.4 Attributable risk fractions of AT on EACs

Table 2 presents the population AFs of non-accidental, cardiovascular, and respiratory EACs in Wuxi associated with non-optimal AT, stratified by sex and age. Overall, the total AF of EACs due to suboptimal AT was 6.88% (95% CI: 4.09–9.26%), with moderate heat contributing the most (3.56, 95% CI: 3.92–10.47%), followed by moderate cold (1.43, 95% CI: −0.11–2.97%), extreme heat (1.20, 95% CI: 0.63 1.81%) and extreme cold (0.75, 95% CI: 0.26 1.25%). The total AF of men (7.34%) and older adults >65 years (7.76%) were higher than that of women (6.26%) and 0–65 years old group (5.43%). Men were more sensitive to moderate colds (AF = 2.10% vs. women, 0.55%). The AF of older adults for moderately and extremely cold conditions was significantly higher than that of the young group. The results showed that mildly high temperature was the most important AT risk factor for EACs, and older adults and men were vulnerable populations with non-optimal AT. For cardiovascular EACs, the total AF was 10.37%, primarily driven by moderate cold (3.22%), with men (16.91%) and older adults (9.88%) at a higher risk. In contrast, respiratory EACs had a lower total AF (4.94%), mainly linked to moderate colds (3.14%), affecting older adults (3.15%) more than younger individuals. Significant AFs were consistently found for moderate and extreme cold conditions concerning cardiovascular and respiratory EACs, whereas heat-related effects showed less pronounced impacts.

**Table 2:** The AFs of non-accidental, cardiovascular and respiratory EACs in Wuxi associated with non-optimal AT, stratified by sex and age.

Variable	All	Moderate heat	Moderate cold	Extreme heat	Extreme cold
Non-accidental EACs	**6.88 (4.09, 9.26)**	**3.56 (1.21, 5.81)**	1.43 (−0.11, 2.97)	**1.20 (0.63, 1.81)**	**0.75 (0.26, 1.25)**
Male	**7.34 (3.92, 10.47)**	3.20 (−0.12, 6.15)	**2.10 (0.02, 4.01)**	**1.15 (0.35, 1.87)**	**0.98 (0.35, 1.63)**
Female	**6.26 (2.62, 9.49)**	**3.99 (0.51, 7.08)**	0.55 (−1.57, 2.76)	**1.23 (0.38, 1.92)**	0.45 (−0.27, 1.13)
0 ~ 65 years	**5.43 (1.42, 8.92)**	3.83 (−0.15, 7.23)	0.08 (−2.25, 2.18)	**1.23 (0.20, 2.09)**	0.23 (−0.54, 0.96)
65 ~ years	**7.76 (4.45, 10.59)**	**3.36 (0.50, 6.09)**	**2.27 (0.20, 4.06)**	**1.16 (0.43, 1.89)**	**1.08 (0.42, 1.74)**
Cardiovascular EACs	**10.37 (5.46, 14.36)**	3.89 (−0.93, 8.13)	**3.22 (1.01, 5.17)**	1.04 (−0.29, 2.23)	**1.33 (0.51, 2.11)**
Male	**16.91 (10.09, 22.58)**	4.46 (−1.42, 10.34)	**7.52 (4.89, 9.99)**	**2.03 (0.26, 3.58)**	**3.19 (2.17, 4.14)**
Female	5.00 (−3.52, 12.07)	2.37 (−4.40, 8.38)	1.05 (−1.76, 3.73)	0.69 (−1.57, 2.51)	0.47 (−0.68, 1.46)
0 ~ 65 years	0.90 (−12.51, 11.94)	−3.63 (−13.34, 5.89)	3.69 (−0.03, 6.85)	−0.84 (−4.44, 1.93)	**1.48 (0.05, 2.74)**
65 ~ years	**9.88 (5.75, 13.50)**	3.73 (−0.29, 7.33)	**3.24 (1.29, 4.98)**	**1.37 (0.31, 2.32)**	**1.41 (0.78, 2.05)**
Respiratory EACs	**4.94 (1.07, 8.76)**	0.09 (−4.04, 3.75)	**3.14(0.96, 5.04)**	0.26 (−0.94, 1.26)	**1.30 (0.54, 2.01)**
Male	**1.06 (0.40, 1.72)**	−0.77 (−6.70, 4.45)	**1.53 (0.49, 2.54)**	−0.46 (−2.29, 1.03)	**0.70 (0.39, 0.98)**
Female	1.70 (−5.17, 7.07)	4.05 (−1.74, 9.13)	−2.52 (−6.73, 1.27)	1.02 (−0.56, 2.43)	−0.79 (−2.28, 0.51)
0 ~ 65 years	−2.62 (−12.6, 5.43)	0.51 (−7.77, 7.93)	−2.18 (−7.99, 2.93)	−0.06 (−2.85, 1.97)	−0.91 (−2.99, 1.15)
65 ~ years	**3.15 (0.75, 5.6)**	0.98 (−0.48, 2.5)	**1.06 (0.18, 2.18)**	0.42 (−0.62, 1.28)	**0.67 (0.14, 0.23)**

AFs, attributable fractions; EACs, emergency ambulance calls; AT, apparent temperature. Bold values indicate *p* < 0.05.

### 3.5 Sensitivity analysis

A sensitivity analysis was performed by varying the degrees of freedom for long-term time trends (6–9) and relative humidity (4–6), assessing alternative lag periods (7 and 21 days), and evaluating potential confounding by NO_2_ and SO_2_ via separate pollutant-adjusted models (Table 3). When these factors were considered with different settings, minimal variation in attributable fractions (AFs) of non-optimal AT on residents’ EACs was observed, indicating that the model was stable.

**Table 3:** Sensitivity analysis of the attributable fraction of apparent temperature on EACs.

Variable	Freedom(f)	AFs (95%CI)		
		Non-accidental EACs	Cardiovascular EACs	Respiratory EACs
Time	6	7.19 (4.42, 9.57)	10.42 (5.52, 14.47)	5.05 (1.22, 8.90)
	7	6.88 (4.09, 9.26)	10.37 (5.46, 14.36)	4.94 (1.07, 8.76)
	8	6.76 (4.01, 9.18)	10.32 (5.44, 14.34)	4.90 (1.01, 8.69)
	9	6.62 (3.87, 9.09)	10.33 (5.45, 14.36)	4.92 (1.05, 8.72)
Relative Humidity	4	6.65 (3.89, 9.11)	10.31(5.39, 14.29)	4.88 (0.98, 8.56)
	5	6.56 (3.74., 8.98)	10.34 (5.42, 14.31)	4.89 (1.02, 8.68)
	6	6.49 (3.69, 8.92)	10.41 (5.50, 14.35)	4.83 (0.92, 8.76)
Lag days	7	6.61 (3.91, 9.11)	10.19 (5.28, 14.19)	4.76 (0.88, 8.56)
	14	6.88 (4.09, 9.26)	10.37 (5.46, 14.36)	4.94 (1.07, 8.76)
	21	6.92 (4.12, 9.31)	10.41 (5.52, 14.41)	4.99 (1.11, 8.83)
+NO_2_	–	6.85 (4.05, 9.22)	10.32 (5.41, 14.30)	4.88 (1.02, 8.70)
+SO_2_	–	6.89 (4.11, 9.27)	10.37 (5.45, 14.39)	4.96 (1.10, 8.79)

AFs, attributable fractions; EACs, emergency ambulance calls; 95CI, 95% Confidence Interval.

## 4 Discussion

This study investigated the effect of non-optimal AT on EACs using daily EAC, meteorological, and air pollution data from Wuxi City between 2014 and 2019. After adjusting for relative humidity, PM_2.5_, O_3_, and long-term trends, a ‘U’-shaped relationship was observed between AT and non-accidental, cardiovascular and circulatory EACs, with the lowest risk AT occurring at 22.54 °C, 17.12 °C, and 12.74 °C, respectively. These findings indicate that both low and high AT levels increase the incidence of non-accidental, cardiovascular, and respiratory EACs in Wuxi residents, which aligns with previous studies reporting varying optimal temperatures for EACs across different regions (28–32). The heterogeneity in optimal temperatures across different studies may stem from multiple contextual factors, including regional climatic characteristics, population demographics, disparities in healthcare resource allocation, urban heat island (UHI) effects, and socioeconomic status.

Our study revealed that high AT exert an immediate effect on EACs, whereas most low AT effects occur with a delay, which is consistent with the results of previous studies (7, 33, 34). The immediate effect of high AT stems from a rapid physiological breakdown dominated by neural regulation, whereas the delayed effects of low AT are closely linked to the cumulative damage mediated by fluid regulation, immune suppression, and metabolic dysregulation (35). However, a study in Zhengzhou found no significant effect of extreme heat (>32.1 °C) on respiratory EACs (29). The observed differences may be attributed to Wuxi’s humid subtropical climate, which exacerbates perceived heat stress during extreme temperature events. This condition impairs the efficiency of respiratory mucosal defence and pathogen clearance. In contrast, Zhengzhou’s semi-arid climate promotes evaporative cooling, enhancing physiological tolerance to heat stress. Furthermore, Wuxi experiences a more pronounced UHI effect resulting from its compact urban form and limited nighttime cooling because of Tai Lake, which prolongs thermal exposure and intensifies acute respiratory impacts.

Males demonstrated significant sensitivity to both heat and cold exposure. Similarly, individuals aged ≥65 years showed a heightened vulnerability to thermal extremes. In contrast, females exhibited a greater susceptibility to extreme heat. These findings align with previous research (8, 9, 36) and may be attributed to physiological and thermoregulatory differences between the sexes. Specifically, females exhibit reduced heat dissipation capacity, resulting in elevated core, skin, and active muscle temperatures. Conversely, males experience more pronounced decreases in core temperature during cold exposure, leading to greater cold intolerance (37, 38). Furthermore, the predominance of males in outdoor labour-intensive occupations, such as agriculture and construction, may contribute to their enhanced heat tolerance through occupational acclimatisation (39). Age-stratified analyses suggested that extreme cold was associated with increased EACs in people aged ≥65 years, which is consistent with the findings of a study conducted in Australia (40). A possible reason for this could be that older adults have poorer physiological regulation capabilities, have more chronic diseases, and may reduce outdoor activities in cold weather, potentially lacking sufficient self-protection awareness or ability (2, 41, 42).

Attribution analysis revealed that for non-accidental EACs, the AF of moderate heat (3.56%) was significantly higher than that of moderate cold (1.20%). This difference stems from two main factors. First, people are more likely to be exposed to heat due to work or daily activities (43, 44) and often lack adequate protective measures, whereas in cold conditions, individuals can mitigate the effects by proper clothing. Second, high temperatures can directly cause acute conditions, such as heatstroke, dehydration, and multi-organ failure, increasing emergency medical demand. This is corroborated by multi-city Chinese data showing a 5.9% AF for heat-related emergency visits (45), with localised studies in Huainan further quantifying a 2.24% AF for ambulance dispatches during extreme heat (46). Similarly, Shenzhen’s findings on heatwave-induced EACs (47) collectively underscore the nationwide heat burden on emergency systems. For cardiovascular EACs, Wuxi’s AF reached 10.37%, which is consistent with Shenzhen’s findings (9.46% for moderate cold stress, particularly affecting males and older adults) (30), highlighting the widespread cardiovascular threat of cold temperatures across China. Our cardiovascular and respiratory EAC analysis revealed that significant AFs were associated with moderate and extreme cold conditions. These findings align with previous studies (45, 47). This phenomenon can be primarily attributed to the prolonged duration of cold weather and its significant and direct effect on the cardiovascular system. Cold temperatures may increase the risk of cardiovascular diseases by influencing the autonomic nervous system, blood pressure, body temperature regulation, inflammatory responses, and oxidative stress (1). In respiratory EACs, a low AT can directly affect respiratory diseases by stimulating airway mucosal vasoconstriction and suppressing immune responses (10). This study has several strengths. First, we used the new composite index AT as an early warning indicator to assess the immediate and delayed effects of temperature on EACs, which may be more realistic and objective than ambient temperature alone. Second, the AFs were calculated to quantify the proportion of EACs attributable to non-optimal AT, thus establishing an evidence base for developing temperature-adaptation strategies in emergency response systems. Finally, we conducted a detailed analysis to compare the effects of AT on different populations of EACs and identify susceptible populations. However, this study has some limitations. First, this study used environmental monitoring station data as the population exposure level for analysis, which belongs to ecological research and may deviate from individual exposure levels. Second, information on medical history, medication use, lifestyle, and access to healthcare services was not included in the emergency care attendance records; therefore, these potential confounders could not be avoided during the analysis. In addition, the lagged effect of temperature on the 120 emergency volumes was only accurate to days and did not consider the variation in hours, which may have led to insufficient accuracy (48). Finally, the limited sample size may have increased the risk of Type II error in some null findings, particularly the observed non-significant association between extreme temperatures and cardiovascular EACs in the 0–65 age group. In future studies, focusing on individual exposure levels and analysing potential risk factors will be necessary to provide a scientific basis for public health protection strategies and measures under extreme temperature conditions.

This study uses historical emergency medical data from Wuxi to provide practical guidance for predicting and preparing for temperature-related emergency burdens. The findings are especially relevant to the Yangtze River Delta region, where cities have similar meteorological conditions, comparable economic development levels, similar healthcare resource allocations, and alike population densities. However, these results may not apply well to other regions in China. Areas with different climate features, like the humid south or arid north, may have different relationships between temperature and emergencies because of varying environmental and socioeconomic factors.

## 5 Conclusion

A non-linear U-shaped relationship was observed between AT and non-accidental, cardiovascular and circulatory EACs, with the lowest risk ATs occurring at 22.54 °C, 17.12 °C, and 12.74 °C, respectively. Both high and low ATs significantly increased the demand for non-accidental EACs, particularly cardiovascular and respiratory EACs. High AT levels have an immediate effect, whereas low levels exhibit a delayed effect. Notably, males and individuals aged ≥65 years were more vulnerable to extreme AT, whereas women demonstrated heightened sensitivity to high AT. These findings are important for developing targeted interventions and hospital service plans to address climate change.

## Data Availability

The processed datasets generated for this study are available on request. The data are not publicly available due to privacy restrictions, but non-identifiable data can be provided by the corresponding author upon reasonable request.
