# Supplementary material for: Associations between apparent temperatures and emergency ambulance calls in Wuxi, China: a time series analysis
Source: Front Public Health. 2025 Sep 9;13:1652961. doi: 10.3389/fpubh.2025.1652961 (PMC12454353; doi:10.3389/fpubh.2025.1652961)
Supplement: Supplementary file 1 [file Table_1.DOCX]

**Associations between apparent temperatures and emergency ambulance calls in Wuxi, China:** **a time series analysis**

Chao Yang ^a, †^, Xiuzhu Li ^a, †^, Wanjun Zhang ^a^, Yiru Tao ^c^, Pengfei Zhu ^a^ , Chuncheng Lu ^b^, Weijie Zhou ^a^, Xinliang Ding ^a, b, *^

^a^ The Affiliated Wuxi Center for Disease Control and Prevention of Nanjing Medical University, Wuxi Center for Disease Control and Prevention, Wuxi Medical Center, Nanjing Medical University, Wuxi, 214023, China

^b^ Key Laboratory of Modern Toxicology of Ministry of Education, School of Public Health, Nanjing Medical University, Nanjing, 211166, China

^c^ Statistics and Information Center of Wuxi Health Commission, Wuxi, 214023, Jiangsu, China

^†^ These authors contributed equally to this work.

* Corresponding author:

Xinliang Ding

The Affiliated Wuxi Center for Disease Control and Prevention of Nanjing Medical University, Wuxi Center for Disease Control and Prevention, Wuxi Medical Center, Nanjing Medical University, Wuxi, 214023, China

[dingxinliang@njmu.edu.cn](mailto:dingxinliang@njmu.edu.cn).

Table S1. RR values between different extreme AT and non-accidental EACs with reference of optimum AT

| Percentile(%) | AT( °C) | RR(95% CI) | | | | |
| --- | --- | --- | --- | --- | --- | --- |
|  |  | Total | Male | Female | 0~65 years | 65~years |
| 2.5 | -1.27 | **1.23 (1.07, 1.41)** | **1.31 (1.09, 1.57)** | 1.13 (0.93, 1.38) | **1.29 (1.03, 1.60)** | **1.21 (1.05, 1.39)** |
| 5 | 0.34 | **1.21 (1.06, 1.38)** | **1.28 (1.08, 1.53)** | 1.13 (0.94, 1.36) | **1.30 (1.05, 1.61)** | **1.18 (1.03, 1.34)** |
| 7.5 | 1.41 | **1.20 (1.06, 1.37)** | **1.27 (1.07, 1.50)** | 1.12 (0.94, 1.35) | **1.31 (1.06, 1.61)** | **1.15 (1.02, 1.31)** |
| 10 | 2.53 | **1.19 (1.05, 1.35)** | **1.25 (1.06, 1.47)** | 1.12 (0.94, 1.34) | **1.31 (1.07, 1.61)** | **1.14 (1.01, 1.28)** |
| 90 | 31.77 | **1.08 (1.03, 1.13)** | **1.08 (1.02, 1.15)** | **1.08 (1.01, 1.15)** | **1.08 (1.02, 1.14)** | 1.10 (0.99, 1.21) |
| 92.5 | 32.98 | **1.10 (1.04, 1.16)** | **1.10 (1.03, 1.19)** | **1.10 (1.02, 1.19)** | **1.11 (1.04, 1.19)** | 1.11 (1.00, 1.24) |
| 95 | 34.18 | **1.13 (1.06, 1.20)** | **1.13 (1.04, 1.23)** | **1.12 (1.03, 1.23)** | **1.14 (1.06, 1.24)** | **1.13 (1.01, 1.26)** |
| 97.5 | 35.98 | **1.17 (1.08, 1.26)** | **1.17 (1.06, 1.29)** | **1.16 (1.04, 1.29)** | **1.20 (1.09, 1.32)** | **1.16 (1.02, 1.31)** |

Note: Abbreviations: RR, relative risk; AT, apparent temperature; EACs, emergency ambulance calls.

Table S2. RR values between different extreme AT and respiratory EACs with reference of optimum AT

| Percentile(%) | AT( °C) | RR (95% CI) | | | | |
| --- | --- | --- | --- | --- | --- | --- |
|  |  | Total | Male | Female | 0~65 years | 65~years |
| 2.5 | -1.27 | **1.36 (1.21, 1.48)** | **1.80 (1.45, 2.25)** | 1.30 (0.88, 1.92) | 1.23 (0.97, 1.55) | **1.47 (1.17, 1.86)** |
| 5 | 0.34 | **1.31 (1.18, 1.41)** | **1.79 (1.44, 2.22)** | 1.26 (0.87, 1.83) | 1.18 (0.96, 1.44) | **1.44 (1.15, 1.80)** |
| 7.5 | 1.41 | **1.25 (1.14, 1.34)** | **1.78 (1.44, 2.19)** | 1.24 (0.87, 1.77) | 1.15 (0.96, 1.37) | **1.41 (1.13, 1.77)** |
| 10 | 2.53 | **1.21 (1.11, 1.28)** | **1.76 (1.43, 2.16)** | 1.21 (0.86, 1.72) | 1.12 (0.95, 1.31) | **1.39 (1.12, 1.73)** |
| 90 | 31.77 | **1.12 (1.03, 1.21)** | **1.14 (1.08, 1.20)** | 1.14 (0.92, 1.42) | 1.36 (0.95, 1.94) | **1.13 (1.06, 1.21)** |
| 92.5 | 32.98 | **1.13 (1.04, 1.24)** | **1.19 (1.12, 1.27)** | 1.18 (0.93, 1.49) | 1.39 (0.96, 2.01) | **1.17 (1.08, 1.26)** |
| 95 | 34.18 | **1.15 (1.05, 1.26)** | **1.26 (1.17, 1.35)** | 1.21 (0.94, 1.56) | 1.43 (0.98, 2.08) | **1.21 (1.11, 1.32)** |
| 97.5 | 35.98 | **1.18 (1.06, 1.31)** | **1.36 (1.24, 1.49)** | 1.27 (0.95, 1.69) | 1.48 (1.00, 2.19) | **1.28 (1.16, 1.42)** |

Note: Abbreviations: RR, relative risk; AT, apparent temperature; EACs, emergency ambulance calls.

Table S3. RR values between different extreme AT and cardiovascular EACs with reference of optimum AT

| Percentile(%) | AT( °C) | RR (95% CI) | | | | |
| --- | --- | --- | --- | --- | --- | --- |
|  |  | Total | Male | Female | 0~65 years | 65~years |
| 2.5 | -1.27 | **1.46 (1.35, 1.68)** | **1.23 (1.06, 1.44)** | 1.32 (0.94, 1.85) | 1.20 (0.72, 2.01) | **1.63 (1.20, 2.20)** |
| 5 | 0.34 | **1.33 (1.26, 1.40)** | **1.20 (1.04, 1.39)** | 1.31 (0.94, 1.82) | 1.22 (0.74, 2.00) | **1.56 (1.16, 2.09)** |
| 7.5 | 1.41 | **1.27 (1.21, 1.34)** | **1.18 (1.03, 1.36)** | 1.30 (0.94, 1.80) | 1.22 (0.75, 1.99) | **1.52 (1.14, 2.03)** |
| 10 | 2.53 | **1.24 (1.18, 1.31)** | **1.16 (1.02, 1.33)** | 1.29 (0.94, 1.77) | 1.23 (0.76, 1.98) | **1.48 (1.11, 1.96)** |
| 90 | 31.77 | **1.26 (1.19, 1.36)** | **1.13 (1.04, 1.23)** | **1.14 (1.04, 1.26)** | **1.26 (1.06, 1.48)** | **1.12 (1.02, 1.23)** |
| 92.5 | 32.98 | **1.35 (1.25, 1.50)** | **1.16 (1.06, 1.27)** | **1.19 (1.06, 1.33)** | **1.33 (1.10, 1.60)** | **1.16 (1.04, 1.28)** |
| 95 | 34.18 | **1.49 (1.29, 1.62)** | **1.19 (1.08, 1.31)** | **1.23 (1.09, 1.40)** | **1.41 (1.14, 1.74)** | **1.19 (1.06, 1.34)** |
| 97.5 | 35.98 | **1.61 (1.46, 1.78)** | **1.24 (1.12, 1.37)** | **1.31 (1.13, 1.52)** | **1.55 (1.20, 2.00)** | **1.26 (1.10, 1.44)** |

Note: Abbreviations: RR, relative risk; AT, apparent temperature; EACs, emergency ambulance call.

Table S4. The single day lag effects(RR with 95% CI) of cold (-1.27℃) and heat (35.98℃ ) at different lag day(s) in non-accidental, cardiovascular and respiratory EACs.

| Lag Days | Non-accidental EACs | | Cardiovascular EACs | | Respiratory EACs | |
| --- | --- | --- | --- | --- | --- | --- |
|  | -1.27℃ vs 22.54℃ | 35.98℃ vs 22.54℃ | -1.27℃ vs 17.12℃ | 35.98℃ vs 17.12℃ | -1.27℃ vs 12.74℃ | 35.98℃ vs 12.74℃ |
| lag0 | 1.007 (0.977, 1.038) | **1.065 (1.036, 1.095)** | 1.031 (0.992, 1.073) | **1.05 (1.012, 1.089)** | 0.989 (0.953, 1.027) | **1.05 (1.013, 1.088)** |
| lag1 | 1.008 (0.988, 1.029) | **1.05 (1.032, 1.068)** | **1.031 (1.004, 1.059)** | **1.038 (1.015, 1.062)** | 0.999 (0.974, 1.024) | **1.036 (1.014, 1.06)** |
| lag2 | 1.01 (0.995, 1.024) | **1.036 (1.025, 1.047)** | **1.03 (1.011, 1.05)** | **1.028 (1.013, 1.042)** | 1.008 (0.99, 1.026) | **1.024 (1.01, 1.038)** |
| lag3 | 1.011 (0.997, 1.026) | **1.023 (1.011, 1.035)** | **1.029 (1.01, 1.048)** | **1.018 (1.003, 1.034)** | 1.015 (0.997, 1.033) | 1.013 (0.998, 1.029) |
| lag4 | 1.012 (0.996, 1.029) | 1.013 (0.998, 1.028) | **1.026 (1.004, 1.048)** | 1.011 (0.991, 1.03) | 1.02 (0.999, 1.041) | 1.006 (0.987, 1.025) |
| lag5 | 1.014 (0.997, 1.03) | 1.006 (0.991, 1.021) | 1.021 (0.999, 1.043) | 1.006 (0.986, 1.025) | **1.022 (1.002, 1.044)** | 1.002 (0.983, 1.021) |
| lag6 | **1.015 (1, 1.029)** | 1.002 (0.989, 1.014) | 1.014 (0.996, 1.034) | 1.003 (0.987, 1.019) | **1.022 (1.003, 1.04)** | 1.001 (0.986, 1.017) |
| lag7 | **1.016 (1.003, 1.028)** | 0.999 (0.989, 1.01) | 1.008 (0.991, 1.025) | 1.002 (0.989, 1.016) | **1.019 (1.003, 1.035)** | 1.003 (0.99, 1.017) |
| lag8 | **1.016 (1.002, 1.031)** | 0.998 (0.986, 1.011) | 1.002 (0.984, 1.021) | 1.002 (0.986, 1.018) | 1.015 (0.997, 1.033) | 1.005 (0.99, 1.021) |
| lag9 | **1.017 (1.001, 1.033)** | 0.998 (0.983, 1.013) | 1 (0.979, 1.021) | 1.003 (0.983, 1.022) | 1.012 (0.991, 1.032) | 1.007 (0.988, 1.027) |
| lag10 | **1.017 (1.001, 1.033)** | 0.997 (0.982, 1.012) | 1.001 (0.98, 1.022) | 1.003 (0.983, 1.022) | 1.01 (0.99, 1.03) | 1.007 (0.988, 1.026) |
| lag11 | **1.017 (1.003, 1.031)** | 0.996 (0.984, 1.007) | 1.006 (0.988, 1.025) | 1.002 (0.986, 1.017) | 1.01 (0.992, 1.028) | 1.004 (0.989, 1.019) |
| lag12 | **1.017 (1.003, 1.031)** | 0.994 (0.984, 1.004) | 1.014 (0.996, 1.033) | 1 (0.987, 1.014) | 1.011 (0.994, 1.029) | 0.999 (0.986, 1.012) |
| lag13 | 1.016 (0.997, 1.036) | 0.992 (0.976, 1.008) | 1.024 (0.999, 1.051) | 0.998 (0.978, 1.02) | 1.013 (0.989, 1.038) | 0.993 (0.973, 1.014) |
| lag14 | 1.016 (0.987, 1.045) | 0.989 (0.964, 1.016) | 1.036 (0.998, 1.075) | 0.996 (0.963, 1.031) | 1.015 (0.98, 1.052) | 0.987 (0.954, 1.021) |

Note: Abbreviations: RR, relative risk; AT, apparent temperature; EACs, emergency ambulance calls.


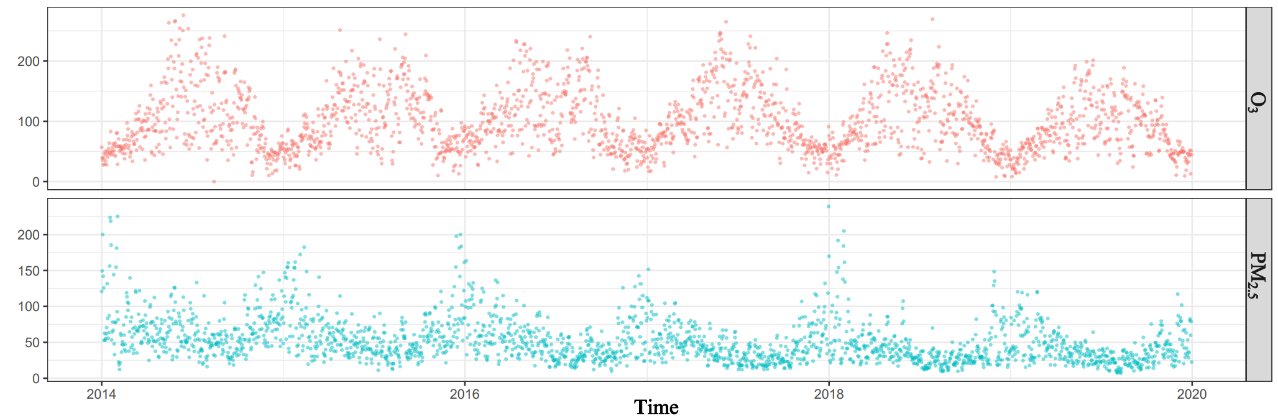

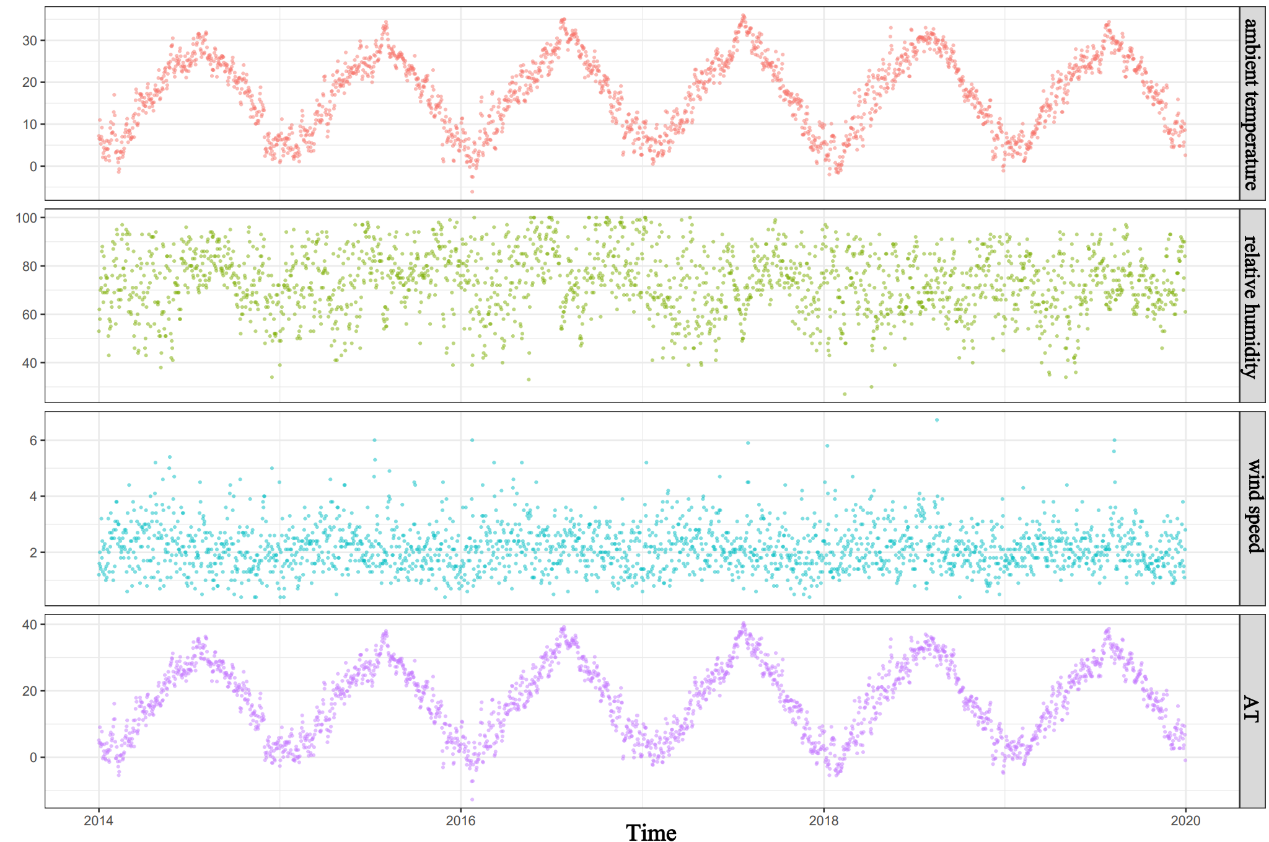


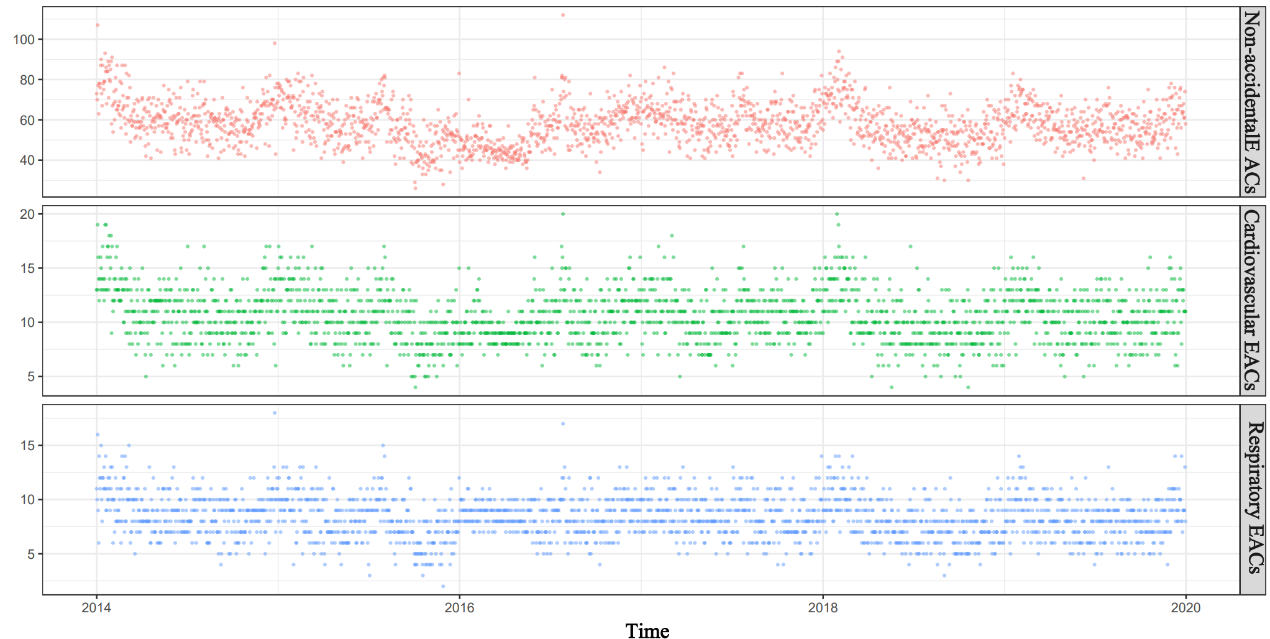


Figure S1. Time-series plots for daily EACs, meteorological variables, O_3_ and PM_2.5_ in Wuxi, China during 2014–2019. Abbreviations: AT, apparent temperature; PM_2.5_, particulate matter (with aerodynamic diameter) less than 2.5 micrometers; O_3_, Ozone.


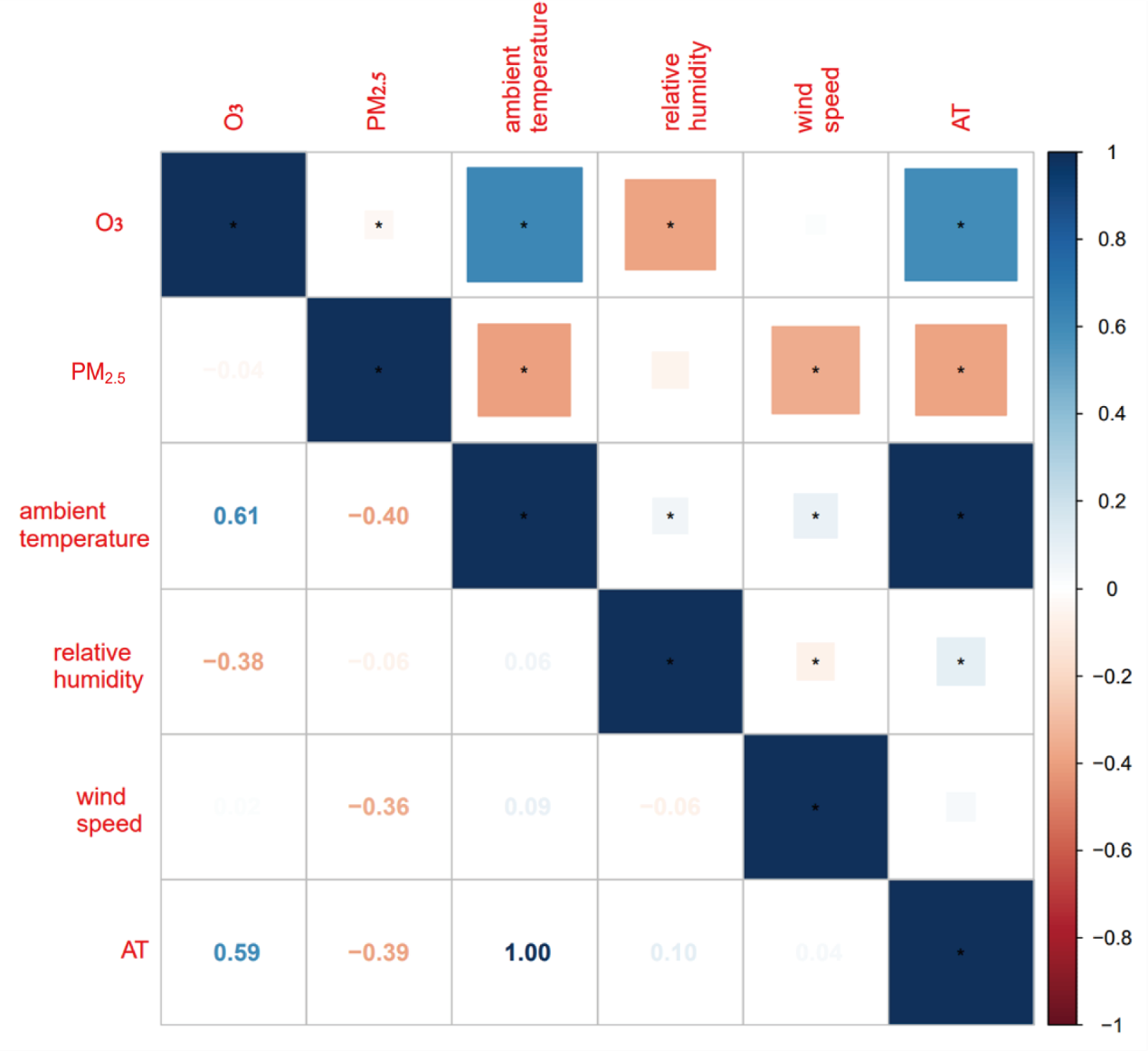


Figure S2. The result of correlation analysis of meteorological factors and air pollutants. * P value＜0.05；Abbreviations: AT, apparent temperature; PM_2.5_, particulate matter (with aerodynamic diameter) less than 2.5 micrometers; O_3_, Ozone.
